# Development of an Efficient Extraction Method for Harvesting Gymnodimine-A from Large-Scale Cultures of *Karenia selliformis*

**DOI:** 10.3390/toxins13110793

**Published:** 2021-11-10

**Authors:** Zhixuan Tang, Jiangbing Qiu, Guixiang Wang, Ying Ji, Philipp Hess, Aifeng Li

**Affiliations:** 1 College of Environmental Science and Engineering, Ocean University of China, Qingdao 266100, China; tangzhixuan@stu.ouc.edu.cn (Z.T.); asttl@ouc.edu.cn (J.Q.); wangguixiang@stu.ouc.edu.cn (G.W.); jy@stu.ouc.edu.cn (Y.J.); 2 Key Laboratory of Marine Environment and Ecology, Ocean University of China, Ministry of Education, Qingdao 266100, China; 3 Ifremer, DYNECO, Phycotoxins Laboratory, F-44000 Nantes, France; Philipp.Hess@ifremer.fr

**Keywords:** *Karenia selliformis*, gymnodimine, liquid-liquid extraction (LLE), extraction method

## Abstract

Gymnodimine-A (GYM-A) is a fast-acting microalgal toxin and its production of certified materials requires an efficient harvesting technology from the large-scale cultures of toxigenic microalgae. In this study the recoveries of GYM-A were compared between several liquid-liquid extraction (LLE) treatments including solvents, ratios and stirring times to optimize the LLE technique for harvesting GYM-A from *Karenia selliformis* cultures, of which the dichloromethane was selected as the extractant and added to microalgal cultures at the ratio 55 mL L^−1^ (5.5%, *v*/*v*). The recovery of GYM-A obtained by the LLE technique was also compared with filtration and centrifugation methods. The stability of GYM-A in culture media were also tested under different pH conditions. Results showed that both the conventional filter filtration and centrifugation methods led to fragmentation of microalgal cells and loss of GYM-A in the harvesting processes. A total of 5.1 µg of GYM-A were obtained from 2 L of *K. selliformis* cultures with a satisfactory recovery of 88%. Interestingly, GYM-A obviously degraded in the culture media with the initial pH 8.2 and the adjusted pH of 7.0 after 7 days, but there was no obvious degradation in the acidic medium at pH 5.0. Therefore, the LLE method developed here permits the collection of large-volume cultures of *K. selliformis* and the high-efficiency extraction of GYM-A. This work provides a simple and valuable technique for harvesting toxins from large-scale cultures of GYM-producing microalgae.

## 1. Introduction

Gymnodimines (GYMs), a group of marine lipophilic toxins with cyclic imine structures, were first detected in oysters from New Zealand [1]. Gymnodimines are characterized by “fast-acting” toxicity, i.e., intraperitoneal injection results in the rapid death of mice [2,3]. So far, more than eight analogs of GYMs have been reported from the microalgal species *Karenia selliformis* and *Alexandrium ostenfeldii*. The dinoflagellate *K. selliformis* mainly produced GYM-A, GYM-B, and GYM-C [4,5], and *A. ostenfeldii* was found to produce 12-methyl GYM-A, 12-methyl GYM-B and GYM-D [6]. Recently, two novel analogs of GYMs, 16-desmethyl GYM-D and GYM-E, were detected in the cultures of *A. ostenfeldii* [7]. Similar to other phycotoxins, a high content of GYMs can accumulate in bivalve mollusks, including in scallops, mussels, oysters, and clams. Shellfish samples contaminated by GYMs have been reported in China [8,9], Europe [10,11], Lebanon [12], Mexico [13] and India [14]. Even though a regulatory limit for GYMs in shellfish has not been adopted due to lack of knowledge regarding their toxicological effects on human health, this toxin group has been evaluated by EFSA [15]. Meanwhile, the EFSA also suggests the development of reference materials for this toxin group. 

Thus, in recent years, the demand for reference materials regarding GYMs has rapidly increased, ranging from exploratory surveys to determine their occurrence in shellfish [10] as well as toxicological and pharmacological research. As known antagonists of nicotinic acetylcholine receptors [16], GYMs may be used as a molecular tool for developing drugs for the treatment of neurodegenerative diseases [17]. While a few studies have reported the successful chemical synthesis of GYMs [18], the synthesis process remains complex and suffers from a low recovery rate. Therefore, extraction from GYM-producing microalgae is still a valuable way to obtain the reference materials of GYMs. For example, the primary supplier of GYM-A-certified reference material, the National Research Council Canada, also purified this toxin from the cultures of *K. selliformis* in the laboratory. However, the GYM production of individual cells of *K. selliformis* was relatively low, resulting in only few tens of pg cell^−1^ [19]. Therefore, some studies explored how to enhance microalgal biomass, such as photobioreactor cultivation [20], the addition of organic acids or trace metals [21,22], and the adjustment of cultural conditions [19]. Meanwhile, the technical challenge has also been raised for toxin collection from large-scale cultures of microalgae. The major techniques that are presently applied in the harvesting of microalgae include centrifugation, flocculation, and filtration [23]. There are common methods for toxin extraction from microalgal cells using organic solvents, such as methanol or acetone. However, harvesting microalgal cells is a time-consuming and laborious task from the large-scale culture, especially for the GYM-producing dinoflagellates that are suspended in the cultures with a relatively low density. In addition, the collection method of adding hydrochloric acid or acetone directly to the cultures to break down the microalgal cells and to release intracellular toxins, followed by the adsorption of toxins by HP20 resin, was also explored [7,24]. This method is also time-consuming and requires a large amount of resin and organic reagents. Currently, a few studies have focused on how to efficiently harvest and extract toxins from large-scale cultures of microalgae. Therefore, the development of a simple, efficient and suitable method for large-scale extraction is essential for the demand of GYMs.

Liquid-liquid extraction (LLE) is widely used to separate target compounds based on relative solubilities in two different immiscible liquids, typically water and organic solvents. With the advantages of a rapid reaction, a high enrichment factor, and simple steps, LLE can be used to separate hydrophobic compounds from the culture medium. The LLE method has been used to separate a wide range of hydrophilic and hydrophobic marine phycotoxins, enabling the comprehensive extraction and analysis of marine toxins [25], in addition to being a rapid pre-treatment method for sample pre-concentration and purification for the detection of marine lipophilic toxins [26,27]. For example, Marrouchi et al. [28] reported the use of dichloromethane and diethyl ether when extracting the GYM toxin in clams using LLE method. To our knowledge, there are no reports using this technique and directly adding extract solvent to the microalgal cultures to recover marine lipophilic toxins. 

It is the aim of this work to develop a simple and efficient LLE method for the extraction of GYM-A from the large-scale cultures of *K. selliformis*. The growth and toxin production at different growth stages of *K. selliformis* as well as the stability of GYM-A in culture medium, were explored in this study. The LLE method was optimized and developed, of which the performance of harvesting GYM-A was also compared with the conventional centrifugation and filtration methods. Finally, the LLE method was adopted to harvest GYM-A from the large-scale cultures of *K. selliformis*.

## 2. Results and Discussion

### 2.1. Growth and Toxin Production of K. selliformis

**Figure 1:**
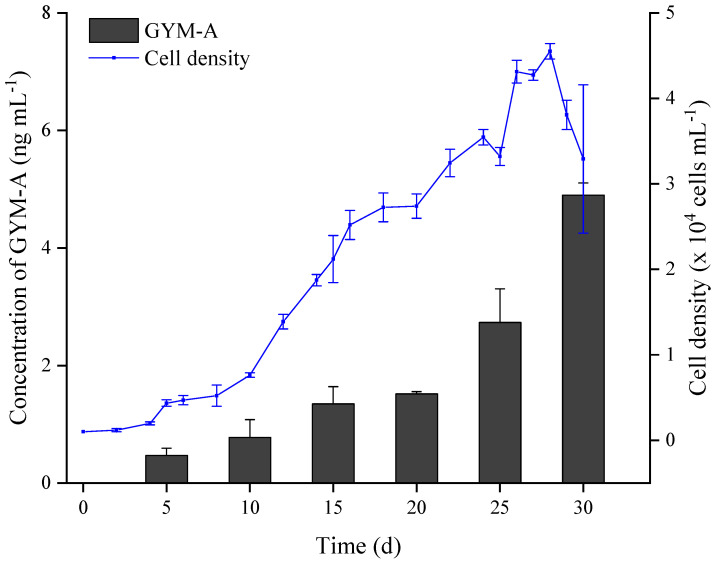
Growth curve and GYM-A production of *Karenia selliformis* during the whole batch culture period. Error bars correspond to the standard deviation (*n* = 3).

The growth curve and toxin production of *K. selliformis* at different growth stages in a batch culture are shown in Figure 1. The growth cycle of GYMs-producing *K. selliformis* strain was about 30 days and it decayed rapidly after day 28. The maximum microalgal density reached 4.6 × 10^4^ cells mL^−1^, which slightly exceeded the cell density obtained in a similar volume of the same strain in a previous study [19]. 

To enrich the extracellular toxins and to reduce the interference of the culture medium matrix on the signal of the LC-MS/MS analysis, HLB SPE cartridges were used to purify the toxins, and the GYM-A recoveries were also evaluated. The GYM-A recoveries at three different concentrations ranged from 99% to 104% (Table S1), demonstrating that the SPE cartridges were suitable for the enrichment and clean-up for GYM-A from the microalgal cultures. 

In this study, filter filtration was used to collect microalgal cells for the analysis of intracellular toxins. However, the microalgal cells were easily fragmented to release toxins during the subatmospheric pressure filtration, which would result in a biased judgement for the distribution of intracellular and extracellular toxins. Therefore, the changes in the total amount of GYM-A in microalgal cultures were evaluated by summing the intracellular and extracellular GYM-A contents. The GYM-A production at different *K. selliformis* growth stage is shown in Figure 1. The total amount of GYM-A increased with the rising of microalgal biomass. The toxin concentration in the microalgal culture system reached 4.9 ng mL^−1^ on the day 30 in the batch culture, suggesting that the cultures should be collected toward the end of the stationary growth phase to obtain a maximum amount of toxins. Additionally, the results (Figure S1) of different *K. selliformis* culture volumes that had been filtered separately onto a piece of a glass-fiber filter showed that GYM-A was mainly distributed inside of the cells. However, a large number of intracellular toxins entered the aqueous phase when the filtered volume of the microalgal cultures was 150 mL or 200 mL (Figure S1), demonstrating that part of the microalgal cells possibly broke and released toxins when cells were exposed to relatively high pressure during the filtration process. The GYM-A quotas of each cell also varied considerably at different growth stages (Table S2). The intracellular toxins content in individual cells was approximately 100 fg cell^−1^ at the initial growth stage (5 and 10 days); the toxin content significantly decreased at the exponential growth stage (15, 20, and 25 days), and finally, the GYM-A production of individual cells recovered and reached 149 fg cell^−1^ (30 days). This phenomenon reflects the typical toxin biosynthesis pattern of dinoflagellates: the intracellular toxin quotas per cell decrease during the exponential growth phase due to the cell mitosis and energy expenditure [29]. While cellular densities were similar to or higher than those obtained using the same strain in a previous study, toxin quotas in our study were much lower than they were in a previous study (5~22 pg cell^−1^), which is up to 150-fold quota of toxins per cell [19]. 

### 2.2. Stability of GYM-A in Culture Medium at Different pH

**Figure 2:**
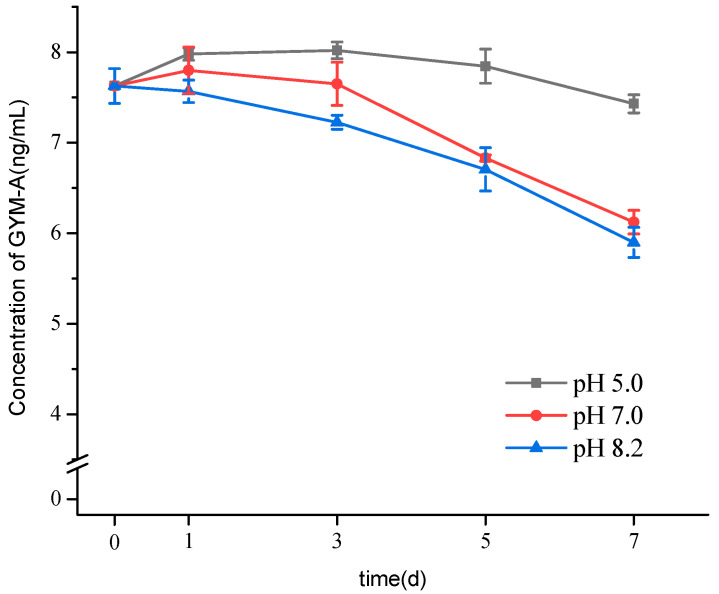
Variations of GYM-A concentrations in the cells-free culture media adjusted by HCl to different pH values.

The variations of GYM-A concentrations in the cell-free culture media of *K. selliformis* under different pH (5.0, 7.0, 8.2) are shown in Figure 2. In the culture media at pH 8.2, 5.3% of the initial content of GYM-A degraded in the first 3-days period. While under neutral conditions, pH 7.0, the average degradation ratios of GYM-A were 10.4% and 19.7% after 5 and 7 days, respectively. However, GYM-A was very stable in the culture media at pH 5.0, and only 2.6% of the initial content of GYM-A were reduced after 7 days. Results showed that GYM-A easily degraded in the cell-free culture media under alkaline and neutral conditions. Beuzenberg et al. [20] also found that GYM-A did not obviously degraded in the culture medium adjusted to pH 5.5 during 15-days storage period. In another previous study, the concentration of GYM-A dissolved in aqueous solution varied depending on the pH conditions at 37 °C, in which GYM-A degraded at pH 7.6 and 8.1, but not at pH 1.8 and 2.8 [3]. Therefore, microalgal cultures should be harvested promptly when the microalgae are in the stationary growth phase to avoid the degradation and loss of GYMs in the culture medium. To store GYM-A toxin, the microalgal cultures should be acidified if they could not be harvested immediately. 

### 2.3. Comparison of Different Techniques for Harvesting GYMs

**Figure 3:**
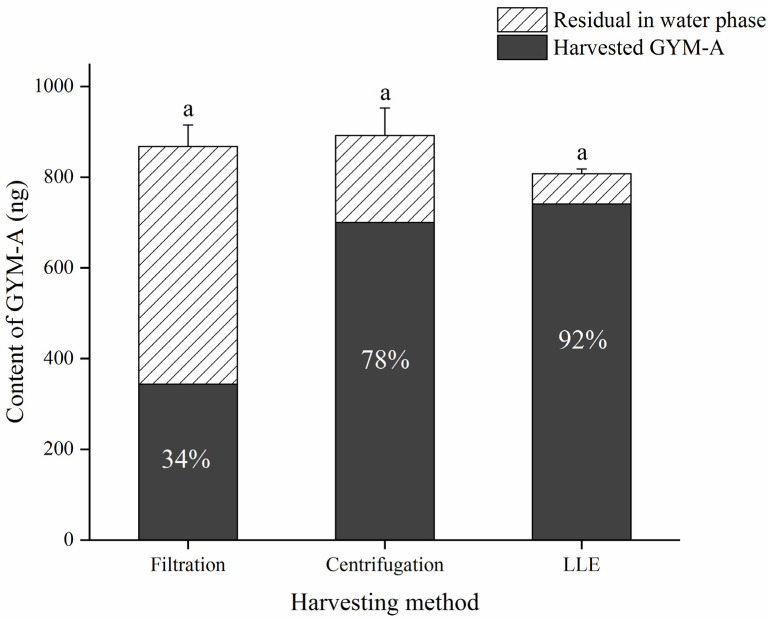
Quantitative results of GYM-A (ng) obtained from the same volumes of *Karenia selliformis* cultures (500 mL) by filtration, centrifugation, and liquid-liquid extraction (LLE) methods. The letter “a” indicates no significant difference between harvesting methods (*p* > 0.05).

The amount and loss of GYM-A collected by different techniques for toxins harvesting are shown in Figure 3. When the microalgal cultures were harvested using the conventional method, filter filtration, only 34% of the toxin content was collected on the filter and about two-thirds of the toxin amount was lost in the solution. This was mainly caused by the genus *Karenia*, which comprises unarmored dinoflagellates with no distinct cell wall plates and that, can be easily fragmented by external force, such as turbulence in the seawater at the surface and at the shore line [30]. Another species of unarmored dinoflagellate, *Karlodinium veneficum* has also been reported to release intracellular karlotoxin during filtration [31]. 

To avoid the broken cells caused by filtration pressure, the microalgal cells were also collected by gentle centrifugation and 78% of the GYM-A amount was retained in the microalgal pellets. Part of the microalgal cells was also fragmented to release intracellular toxins during the centrifugation process. Therefore, it can be identified that the *K. selliformis* cells were fragmented at different degrees during the harvesting processes of filter filtration and centrifugation methods. In a previous study, non-negligible toxin loss was also found when *Azadinium spinosum* was harvested by tangential flow filtration, even though this technique is not considered to be harsh on cells [32]. 

Aromatic HP20 resin was used to adsorb the GYMs from the culture medium comprising the fragmented *K. selliformis* or *A. ostenfeldii* cells due to its ability to adsorb toxins from the seawater [7,24]. Additionally, HP20 resin was also applied to enrich other lipophilic toxins (AZA1, AZA2, OA, DTX1) that had been released into the culture medium [32,33]. In this study, SPATT bags containing HP20 resin were placed in the culture medium to absorb GYM-A for 24 h in order to evaluate its adsorption capacity. However, resin bags were saturated after 12 h of adsorption, and 58% of the GYM-A remained in the filtrate solution after 24 h (Figure S2). Therefore, the performance of SPATT method for GYM-A could not support it as a potential utility for harvesting this toxin. 

The liquid-liquid extraction (LLE) method is commonly applied for sample separation and preconcentration, but it has not been used for direct extraction of microalgal cultures. Satisfactory recovery of GYM-A was obtained by the LLE method from the culture media in this study. The dichloromethane extraction was able to rapidly fragment *K. selliformis* cells to release intracellular toxins, in which the GYM-A was subsequently partitioned into the organic phase. 

The costs of the above harvesting methods in terms of organic solvents, time and economy are shown in Table 1. As an environmentally friendly method, HP20 resin can enrich toxins and reduce the use of organic solvents, but it is time-consuming and inefficient and has a low recovery rate (42%). In addition, it cannot be ignored that the loss of GYM-A in the culture media due to the time-consuming procedures. Although filtration or centrifugation combined with resin adsorption can also be used to collect toxins from microalgal cultures, these methods involve several processes such as microalgal cell collection, resin adsorption, and toxin extraction, which are relatively time consuming. Especially, the time cost of filtration and centrifugation are directly proportional to the volumes of harvested microalgae, but the LLE method can reduce the time consumption by scaling up the reaction. In addition, the LLE method integrates microalgal culture collection and toxin extraction with a high recovery and the relatively simple operation and does not require the use of additional instruments such as vacuum filter, ultrasonic cell disruptor, liquid nitrogen tanks, etc. Therefore, the LLE method is more suitable for harvesting large volumes of *K. selliformis* cultures to extract GYMs. We speculate that the LLE technique could be potentially applied to other unarmored toxin-producing dinoflagellates, if the suitable extraction conditions are optimized. 

### 2.4. Optimization of Liquid-Liquid Extraction Method

#### 2.4.1. Extraction and Dispersion Solvents

**Figure 4:**
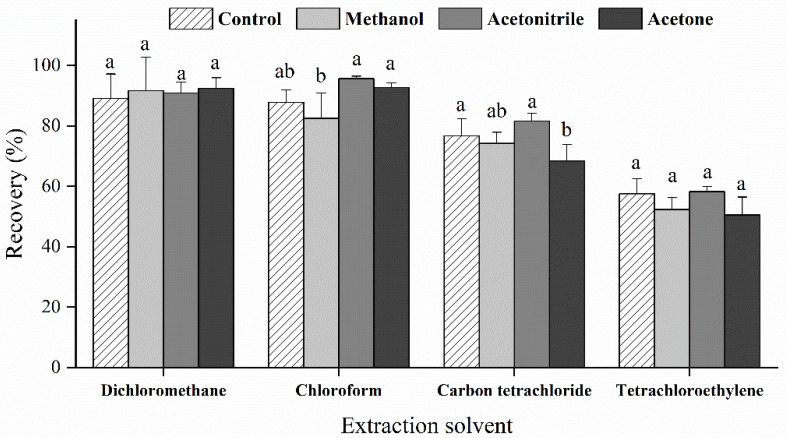
Extraction recovery of GYM-A (%) in the liquid-liquid extraction method adopted by different combinations of extraction and dispersion solvents (control group without dispersion solvent). Different letters (a, b, ab) indicate significantly different values at *p* < 0.05.

To obtain good selectivity and extraction efficiency for a target compound, it is a prerequisite to select an appropriate extraction solvent. The extraction solvents should have the characteristics of low water solubility, high extraction efficiency, and a large density difference with water. The dispersion solvent is also crucial for the extraction procedure, which should have good miscibility in both the extraction solvent and the aqueous phase. It can promote the extraction solvent to be highly dispersed in the aqueous phase, thus increasing the interaction area, which greatly accelerates the exchange of the target compound between the two phases and improves the extraction efficiency [34]. The extraction efficiency of GYM-A under different combinations of extractant and dispersant is shown in Figure 4. No significant differences were observed in the extraction recoveries with or without a dispersion solvent (*p* > 0.05), except for the combination of carbon tetrachloride and acetone. Furthermore, in the absence of dispersing solvents, the recoveries of GYM-A in the dichloromethane and chloroform groups were 89% and 88%, respectively, which were more efficient than the extraction of the carbon tetrachloride (77%) and tetrachloroethylene (58%) groups. It is speculated that a small portion of GYM-A was dissolved and dispersed in seawater due to the presence of a salt substrate. The dispersant can accelerate the exchange of the target compound between the two phases and can cause the extraction reach equilibrium more quickly, but was not able to significantly improve the extraction recovery due to the good dissolution of GYM-A in seawater in the present study. Therefore, the dispersed solvent can be discarded in the LLE procedure and the satisfactory recovery of GYM-A could still be obtained without these solvents. Good recovery of GYM-A in the spiked seawater was also achieved using chloroform as an extraction solvent in an optimized dispersive liquid-liquid microextraction by Oller-Ruiz et al. [27]. In some previous studies, dichloromethane was also used as an extractant to extract GYM from digestive glands and microalgae [4,28]. Thus, dichloromethane was the most suitable extraction solvent for GYM-A used in this study because of its high extraction efficiency, low cost, and relatively low toxicity compared to other chlorinated solvents.

The pH of the sample can also affect the extraction efficiency. It was found that an acidic pH favored the extraction of acidic toxins (PTX2, DTXs and OA), while an alkaline pH favored the extraction of other toxins (AZAs, SPXs and GYM) during dispersive liquid-liquid microextraction [27]. In this study, a satisfactory GYM-A extraction efficiency was achieved in the cultures of the initial seawater pH, and the effect of pH on extraction was not considered any further. It should be noted that GYM-A is susceptible to degradation under alkaline conditions, so the toxin collection or acidification of the microalgal cultures should be performed as soon as possible. 

#### 2.4.2. Volume of Dichloromethane

**Figure 5:**
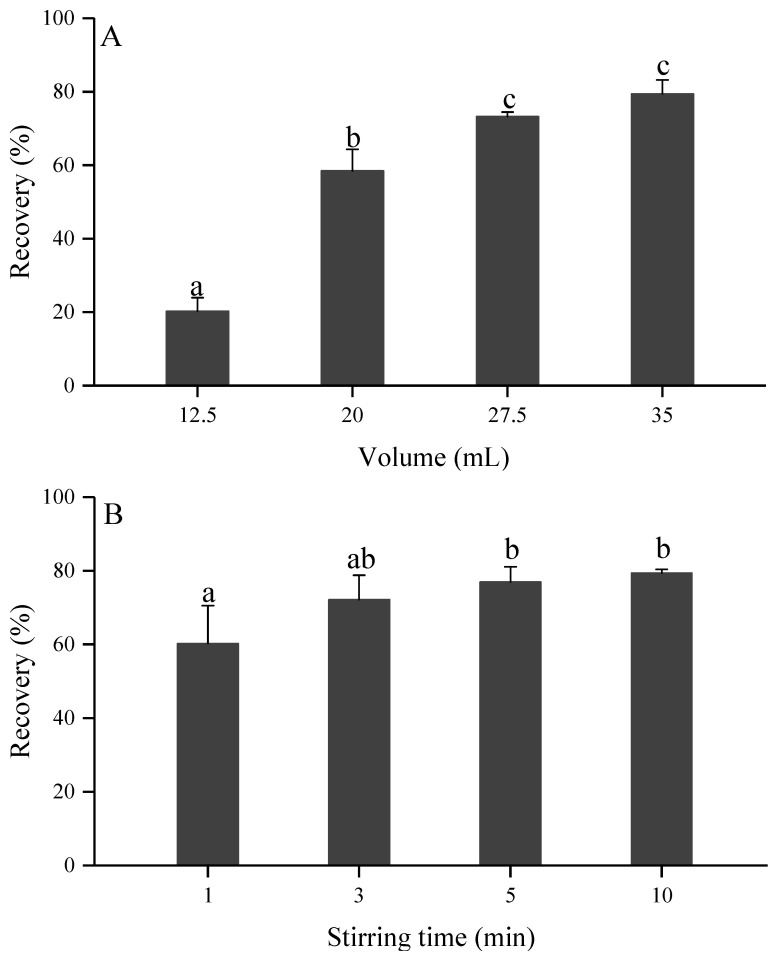
GYM-A (%) recovery obtained by the liquid-liquid extraction method with different volumes of dichloromethane (**A**) and magnetic stirring times (**B**). Different letters (a, b, c, ab) indicate significantly different values at *p* < 0.05. Treatments with different letters are statistically significantly different. The “ab” treatment is not different from either “a” or “b” treatments.

The results of the GYM-A extraction efficiency achieved by LLE method with the addition of different volumes of dichloromethane to 500 mL of *K. selliformis* cultures are shown in Figure 5A. The results showed that the extraction efficiency increased when the volume of dichloromethane increased. The GYM-A recoveries were as low as 20% and 58% when the additive volumes of dichloromethane were 12.5 mL and 20 mL, respectively, which possibly resulted from the fact that the small amounts of dichloromethane that were added to the seawater did not completely destroy the microalgal cells. A relatively high recovery of 73% was obtained when the dichloromethane additional volume reached 27.5 mL and it was not significantly improved in the treatment group with 35 mL of dichloromethane (*p* > 0.05). We hypothesized that the dichloromethane addition ratio in the microalgal cultures of 55 mL L^−1^ (27.5 mL dichloromethane/0.5 L culture) was enough to completely lyse the microalgal cells to release GYM-A in the extraction process. Thus, the optimal ratio of dichloromethane to the microalgal cultures was established at to be 55 mL L^−1^ to save chemical reagents and protect environment. 

#### 2.4.3. Stirring Time

The formation of small droplets of dichloromethane during the stirring process was able to increase the interaction surface area between the extraction solvent and the aqueous phase, enabling a rapid exchange of the target compound. It is generally expected that the extraction reaction will reach equilibrium after a very short amount of time, but the allotted time must ensure that the microalgal cells are completely ruptured in order for them to release the toxins. The effects of different stirring times on the extraction efficiency are shown in Figure 5B. The extraction recovery of GYM-A was 60% in the 1 min stirring group, which was significantly lower than that in the 5 min and 10 min stirring treatment groups (*p* < 0.05). The extraction recoveries of GYM-A were 72%, 77% and 79% in the 3 min, 5 min and 10 min stirring treatment groups, respectively, without any significant differences (*p* > 0.05) being observed. The results showed that stirring for 1 min could not completely break down the microalgal cells and that the extraction reaction was not enough. Based on the changes to the mixture color during the stirring process, it could also be inferred that the microalgal cells would gradually break up and release intracellular compounds within 1 to 3 min of stirring. Considering the extraction efficiency and ability to save time, 5 min was chosen as the optimal stirring time in this study.

**Figure 6:**
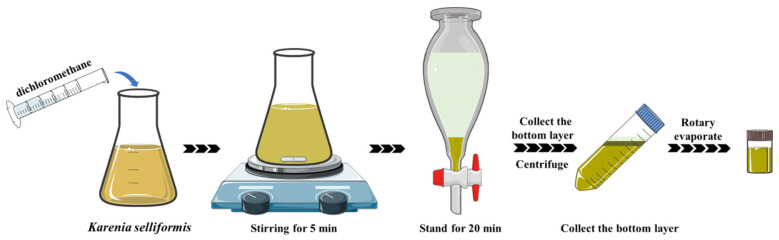
Schematic diagram of the liquid-liquid extraction (LLE) developed in this study.

In summary, the final optimized LLE method was operated as follows (Figure 6): dichloromethane, was added to the *K. selliformis* cultures as the extraction, with the volume ratio of dichloromethane to cultures of 55 mL L^−1^. The mixture was magnetically stirred for 5 min at 2400 rpm, and it was then transferred to a partition funnel and was left to stand for 20 min. The organic phase was collected, and it was then centrifuged at 4750× *g* for 10 min. The organic phase was collected, then dichloromethane was added to the centrifuge tube and it was centrifuged again. The organic phases were combined and were rotary evaporated, and the residue was reconstituted in MeOH.

### 2.5. Application of the Optimized Liquid-Liquid Extraction Method

The optimized LLE method was applied to collect GYM-A from 2 L of *K. selliformis* cultures. A total of 5.1 μg GYM-A were collected from the microalgal cultures with a recovery of 88 ± 3.5%. The LC-MS/MS chromatograms of GYM-A in the crude GYM-A extracts are shown in Figure S3. This application demonstrated that the optimized LLE method can be applied to the collection of toxins from the large-scale volume of microalgal cultures before purifying certified reference materials. 

## 3. Conclusions

In this study, the growth and toxin production of *Karenia selliformis*, and the stability of GYM-A in culture medium were investigated. For the first time, a novel and simple liquid-liquid extraction (LLE) technique was developed and optimized for the rapid harvesting GYM-A from the *K. selliformis* cultures. Compared to conventional centrifugation and filtration methods, LLE has better applicability and offers several advantages such as a simple extraction procedure, low cost, time savings, and satisfactory recovery. The optimized LLE method can be applied to harvest GYM-A from large volumes of *K. selliformis* cultures. This work will be useful for the preparation of GYM-A reference materials and may also serve as a guide for the large-scale extraction of other active substances from microalgae.

## 4. Materials and Methods

### 4.1. Chemicals

Dichloromethane, chloroform, carbon tetrachloride, and tetrachloroethylene were purchased from Macklin Ltd. (Shanghai, China). Acetonitrile, methanol (MeOH), and acetone were obtained from Merck Ltd. (Darmstadt, HE, Germany). Sodium hydroxide, ammonium hydroxide (NH_4_OH), and hydrochloric acid (HCl) were purchased from Fisher Scientific (Fair Lawn, NJ, USA). All chemical reagents were of HPLC-grade purity. The GYM-A certified reference material (CRM) was purchased from the National Research Council (Halifax, NS, Canada). Milli-Q water (18.2 MΩ cm resistivity or better) was obtained from a Milli-Q water purification system (Millipore Ltd., SAS., Molsheim, France). The adsorbent resin, HP20, was purchased from the Mitsubishi Chemical Corporation (Tokyo, Japan).

### 4.2. Culture of Microalgae and Toxin Production

The GYM-A producing strain of *K. selliformis* (GM94GAB) used in this study was isolated from the Gulf of Gabes, Tunisia [19]. The *K. selliformis* culture method was described in our previous study [8]. In brief, the strain was cultured in sterile-filtered (0.45 μm membrane, Xingya, Shanghai, China) seawater before enrichment with an f/2 medium without silicate. The culture was kept at 18 ± 2 °C under a photon flux density of 111 μmol m^−2^ s^−1^ with a 12 h light: 12 h dark cycle.

The *K. selliformis* strain was cultured for 30 days from an initial density of 1000 cells mL^−1^ in 21 conical flasks containing 400 mL of culture medium. The culture flasks in the illumination incubator were randomly moved every day to avoid any difference in the light intensity. Samples (1 mL) were taken from the culture to count the cell density by optical microscope every two days to obtain a growth curve. In addition, three conical flasks were randomly sampled every five days to determine the intracellular and extracellular toxin contents. Cultures were filtered with a GF/F filter (Whatman, pore size 0.7 µm, diameter 47 mm) and the subatmospheric pressure was between 0.07 and 0.08 MPa during the filtration process. The filter and culture medium (50 mL) were used to analyze the intracellular and extracellular toxins, respectively.

### 4.3. Extraction of Toxins

#### 4.3.1. Microalgal Cells

The filtered GF/F filter was cut into small pieces, and the pieces were placed into a 10 mL centrifuge tube, and then 3 mL MeOH were added and mixed for 1 min. The centrifuge tube was then freeze-thawed three times in liquid nitrogen for 5 min per cycle. The mixture was then sonicated by a sonication probe (KS-750F, Kesheng Ultrasonic Equipment Ltd., Ningbo City, China) in an ice bath for 10 min, and then the mixture was centrifuged at 5581× *g* for 10 min. The supernatant was transferred to a 10 mL volumetric flask. The pellet was extracted twice more with an additional 3 mL of MeOH. All of the extracts were combined and brought up to 10 mL with MeOH. Subsamples (1 mL) were filtered (0.22 μm organic filter, Peak Sharp, Beijing, China) into a vial and were stored at −20 °C until LC-MS/MS analysis.

#### 4.3.2. Culture Medium

Extracellular toxins in culture medium were enriched by an Oasis^®^ HLB SPE cartridge (3 cc, 60 mg, Waters, Medford, MA, USA) as described in our previous study [8]. The cartridge was activated and conditioned with 100% MeOH (3 mL) and 20% MeOH (3 mL), respectively. Then, 12.5 mL of MeOH were added to 50 mL of culture medium to make a 20% MeOH (*v*/*v*), and the mixture was loaded onto a cartridge. An amount of 3 mL of 20% MeOH were used to wash the cartridge and 3 mL of 100% MeOH were used to elute the GYM toxins. The volume of the eluate was brought up to 5 mL with MeOH. A subsample of the eluate was filtered and stored using the same method as the one described above.

### 4.4. Stability of GYM-A in the Culture Medium with Different pH

Culture medium of *K. selliformis* at the stationary growth phases was collected by centrifugation at 2500× *g* for 5 min. The culture medium with an initial pH of 8.2 was adjusted to 5.0 and 7.0 with 6 mol L^−1^ of hydrochloric acid. Culture media (200 mL) with the three different pH values (5.0, 7.0, 8.2) were placed into nine conical flasks for seven days at 20 °C. Subsamples (10 mL) were collected and extracted using HLB SPE cartridges (3 cc, 60 mg) to determine the GYM-A concentration after 1, 3, 5, and 7 days, respectively.

### 4.5. Comparison of Different Toxin Harvesting Methods

The filter filtration, centrifugation, and liquid-liquid extraction (LLE) performances was compared for harvesting the GYMs. Triplicate microalgal cultures (500 mL) were collected by either filtration, centrifugation, or LLE. 

The extraction of GYM-A from the *K. selliformis* cells harvested by filtration was conducted using the same procedures described above, and the filtrated toxin (20 mL) was enriched by HLB SPE cartridges (3 cc, 60 mg).

Cultures were centrifuged at 2500× *g* for 5 min, and the pellets and supernatant were collected. The supernatants (20 mL) were treated by HLB SPE cartridges (3 cc, 60 mg) to obtain the extracellular toxins. The pellets were suspended in 10 mL of MeOH and the same extraction method as the one described in Section 4.3.1 was used. The pellet was extracted twice more with the addition of 5 mL MeOH, and then the extracts were combined and brought up to 25 mL with MeOH. An aliquot of 1 mL of extract was filtered through a 0.22 μm filter into a glass vial for LC-MS/MS analysis.

Cultures (500 mL) were mixed with 27.5 mL dichloromethane. After thorough mixing by magnetic stirring at 2400 rpm for 5 min, the mixture was transferred to a separatory funnel and was left to stand for 20 min. The lower layer was collected and centrifuged at 4750× *g* for 10 min and was divided into three parts: aqueous phase, residue and dichloromethane. The dichloromethane was then transferred to a sample bottle and the remainder was re-extracted by 5 mL dichloromethane. The dichloromethane was combined and dried by a gentle stream of nitrogen, and the residue was reconstituted in 5 mL of 100% MeOH. Subsamples (1 mL) were filtered through a 0.22 μm organic filter for LC-MS/MS analysis.

HP20 resin adsorption for GYM-A was also tested in cell-free culture medium. Solid phase adsorption toxin tracking (SPATT) bags with 1.0 g of dry HP20 resin was treated as described in our previous study [35]. After pretreatment, the bag was stirred continuously at 145 rpm in culture medium (1 L) and 10 mL of sample were taken at 0 h, 1 h, 3 h, 6 h, 12 h, and 24 h, and were purified using HLB SPE cartridges (3 cc, 60 mg) to determine the concentration of the GYM-A in the culture medium.

### 4.6. Optimization of Liquid-Liquid Extraction

#### 4.6.1. Extraction and Dispersion Solvents

The GYM-A certified reference material was dried with nitrogen and reconstituted to a concentration of 0.5 ng mL^−1^ in filtered (0.45 μm) seawater. The seawater subsample (1.0 mL) was taken and purified by HLB SPE cartridge (3 mL, 60 mg) to determine the initial GYM-A concentration. Dichloromethane (CH_2_Cl_2_), chloroform (CHCl_3_), carbon tetrachloride (CCl_4_) and tetrachloroethylene (C_2_Cl_4_) were selected as the extractants, and methanol, acetonitrile, and acetone were selected as dispersants. The extraction solvent (200 μL) and dispersion solvent (100 μL) were rapidly added to 5 mL of the seawater sample spiked with GYM-A. Triplicate control treatments without dispersion solvents were used separately in four different experimental groups. The sample was vortexed for 2 min and was then centrifuged at 1645× *g* for 5 min to separate both phases. The upper aqueous phase was removed by a glass pipette, and the lower organic phase was evaporated under a nitrogen blow. The residual material was reconstituted with 1.0 mL of MeOH, filtered (0.22 μm), transferred to a glass vial and stored at −20 °C until analysis. Recovery was calculated by the equation, Recovery (%) = *C_e_*/*C_0_* × 100%, where *C_e_* corresponds to the GYM-A content collected by liquid-liquid extraction, and *C_0_* represents the initial GYM-A content in the seawater sample. 

#### 4.6.2. Dichloromethane Volume

The *K. selliformis* cultures were collected in the stationary growth phase. Microalgal cultures (10 mL) were taken and centrifuged at 2500× *g* for 5 min. The target GYM-A was extracted from the supernatants and pellets as described above, and the initial toxin concentration was determined. Microalgal cultures (500 mL) were placed into 12 conical flasks, which were divided into four groups. In each group, 12.5 mL, 20 mL, 27.5 mL and 35 mL of dichloromethane were added, respectively. Liquid-liquid extraction was performed as described above. Subsamples (1 mL) were filtered through a 0.22 μm organic filter for LC-MS/MS analysis. The liquid-liquid extraction recovery was determined by the ratio of the extracted and the initial GYM-A content. 

#### 4.6.3. Stirring Time

Microalgal cultures (500 mL) were mixed with 27.5 mL dichloromethane. The mixture was then magnetically stirred at 2400 rpm for 1 min, 3 min, 5 min and 10 min. The following extraction process was carried out using the same procedures as the ones described above.

### 4.7. Application of LLE for Toxin Collection from Microalgal Cultures

The optimized LLE method was applied to the GYM-A collection from the *K. selliformis* cultures, and the LLE schematic procedure is shown in Figure 6. Dichloromethane (110 mL), was added to the cultures of *K. selliformis* (2 L) as the extract solvent at a ratio of 55 mL L^−1^. The same liquid-liquid extraction process as the ones described above was used to harvest the toxins. The collected dichloromethane phase was dried by rotary evaporation, and the residue was reconstituted in MeOH. 

### 4.8. LC-MS/MS Analysis

An Ultimate 3000 HPLC (Thermo Fisher Scientific, Bremen, Germany) equipped with an AB-Sciex Qtrap 4500 mass spectrometer (AB Sciex Pte. Ltd., Singapore) using an ESI interface was used to analyze all of samples. An X-Bridge^™^ C18 column (150 × 3 mm, 5 µm, Waters, Milford, MA, USA) was used to separate GYM at 35 °C. The mobile phase was composed of solvent A (water) and solvent B (90% acetonitrile), each containing 6.7 mmol L^−1^ NH_4_OH. A gradient was run at 300 μL min^−1^ from 50% to 100% B over 8 min, held for 1 min and returned to 50% B over 1 min before re-equilibration for 1 min for the next run. The injection volume was set at 5 μL. Multiple reaction monitoring (MRM) with the positive ionization mode was used to qualify and quantify GYM-A (*m*/*z* 508.3 > 490.3/392.2/162.1). The limit of detection (LOD) of GYM-A was 4.8 pg mL^−1^, and the linear range of GYM-A was 4.8 to 13,000 pg mL^−1^.

### 4.9. Data Processing

Data were expressed as mean ± standard deviation (SD). One-way analysis of variance (ANOVA) followed by the Least Significant Difference (LSD) test was employed to identify the significant differences (*α* = 0.05) using SPSS statistical package version 25 (IBM corporation, Armonk, NY, USA). All figures were drawn by the software Origin 2017 (Origin Lab, Hampton, MA, USA).

## Figures and Tables

**Table 1:** Comparison of different harvesting methods for 500 mL of *K. selliformis* cultures.

Extraction Method	Operation Process	Solvent Cost	Time Cost	Economic Cost (RMB)	Recovery (%)
HP20 resin adsorption	resin activation 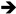 adsorption 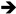 toxin desorption	10 mL methanol	1 day	3.00	42
Filtration	suction filtration 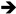 filter cut off 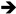 freeze-thaw 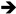 probe sonication	30 mL methanol	50~60 min	25.05	34
Centrifugation	centrifugation 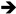 freeze-thaw 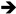 probe sonication	20 mL methanol	30~40 min	1.10	78
LLE	liquid-liquid extraction 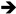 stand	27.5 mL dichloromethane	25 min	1.07	92

## Data Availability

The data in this study are available in this article and Supplementary Materials.

## Supplementary Materials

The following are available online at https://www.mdpi.com/article/10.3390/toxins13110793/s1, Figure S1: GYM-A distribution in algal pellets on filter and aqueous phase when collecting different volumes of *K. selliformis cultures* by filter filtration, Figure S2. Variation of GYM-A content (%) in *K. selliformis* filtrate with adsorption time of HP20 resin bag, Figure S3. LC-MS/MS chromatograms of GYM-A in standard (A) and crude GYM-A methanolic extracts (B), Table S1: Recovery (%) of spiked GYM-A with different concentrations (ng mL^−1^) in seawater loading on HLB SPE cartridge, Table S2: Intracellular GYM-A content in different growth stages of *Karenia selliformis* (fg cell^−1^).
