# Supplementary material for: Development of an Efficient Extraction Method for Harvesting Gymnodimine-A from Large-Scale Cultures of Karenia selliformis
Source: Toxins (Basel). 2021 Nov 10;13(11):793. doi: 10.3390/toxins13110793 (PMC8621799; doi:10.3390/toxins13110793)
Supplement: Supplementary file 1 [file toxins-13-00793-s001.zip › toxins-1430754-supplementary.pdf]

toxins

MDPI

Toxins **2021**, *13*, 793

Supplementary Materials: Development of an Efficient Extraction Method for Harvesting Gymnodimine-A from Large-Scale Cultures of *Karenia selliformis*

Zhixuan Tang, Jiangbing Qiu, Guixiang Wang, Ying Ji, Philipp Hess and Aifeng Li

Table S1. Recovery (%) of spiked GYM-A with different concentrations (ng mL^{-1}) in seawater loading on HLB SPE cartridge.

Spiked concentration	Recovery	RSD
97	99	2.0
10	101	1.8
1.1	104	1.3

Table S2. Intracellular GYM-A content in different growth stages of *Karenia selliformis* (fg cell^{-1}).

Growth time (day)	5	10	15	20	25	30
Intracellular GYM-A	107	102	64	55	82	149

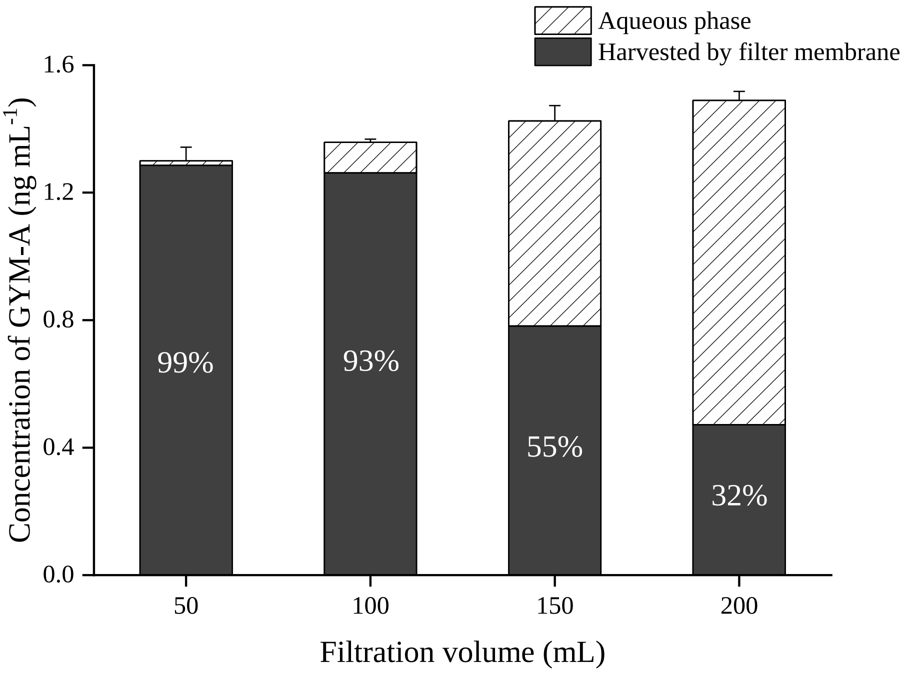

Figure S1. GYM-A distribution in algal pellets on filter and aqueous phase when collecting different volumes of *K. selliformis cultures* by filter filtration.

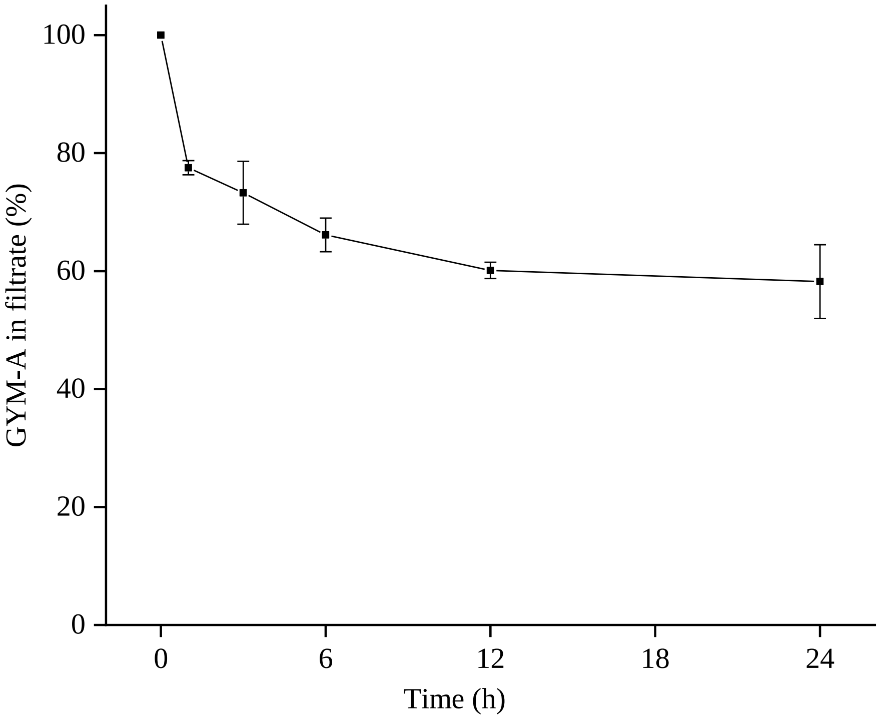

Figure S2. Variation of GYM-A content (%) in *K. selliformis* filtrate with adsorption time of HP20 resin bag.

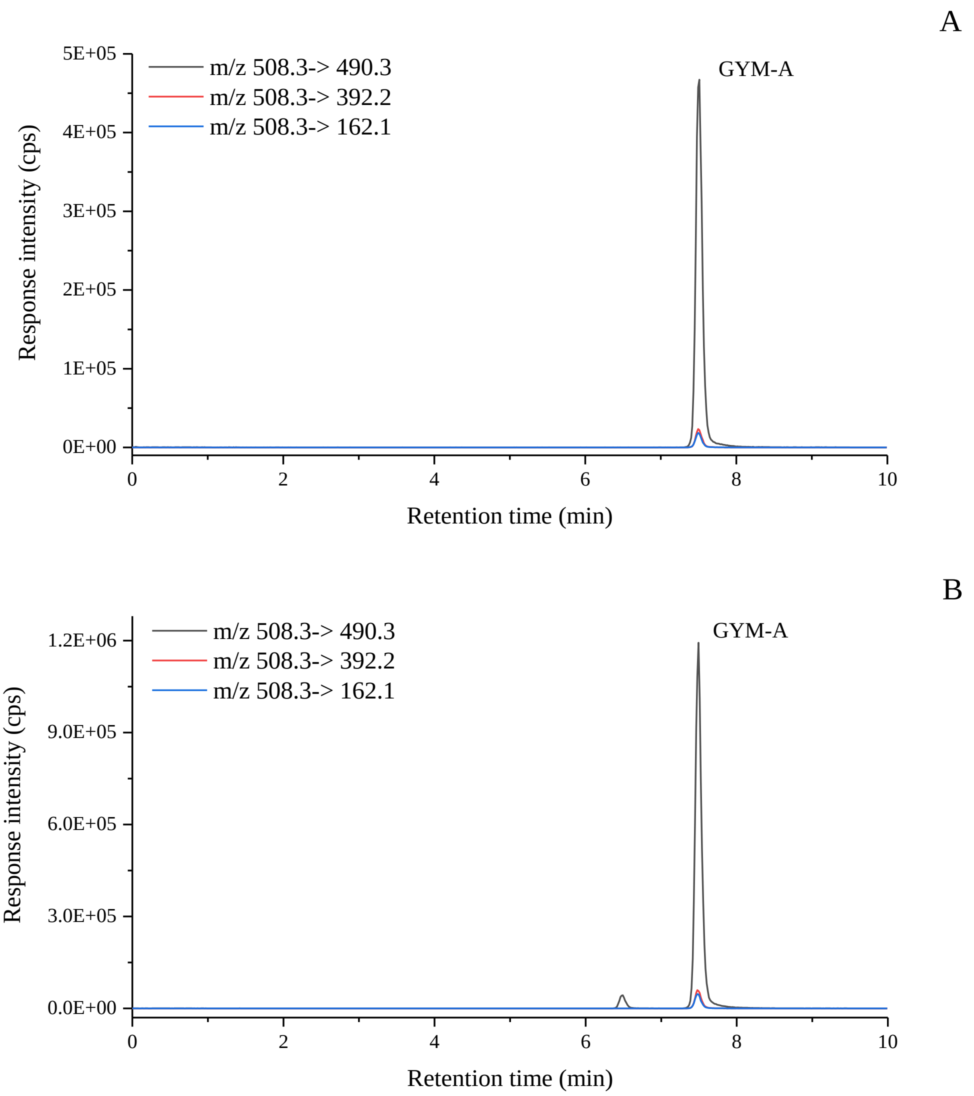

Figure S3 LC-MS/MS chromatograms of GYM-A in standard (A) and crude GYM-A methanolic extracts (B).-positive MRSA.
